# Novel candidate genes influencing natural variation in potato tuber cold sweetening identified by comparative proteomics and association mapping

**DOI:** 10.1186/1471-2229-13-113

**Published:** 2013-08-07

**Authors:** Matthias Fischer, Lena Schreiber, Thomas Colby, Markus Kuckenberg, Eckhard Tacke, Hans-Reinhard Hofferbert, Jürgen Schmidt, Christiane Gebhardt

**Affiliations:** 1 Department of Plant Breeding and Genetics, Max-Planck-Institute for Plant Breeding Research, Cologne, Germany; 2 Max-Planck-Institute for Plant Breeding Research, Mass Spectrometry Group, Cologne, Germany; 3 BIOPLANT, Biotechnologisches Forschungslabor GmbH, Cologne, Germany; 4 Böhm-Nordkartoffel Agrarproduktion OHG, Ebstorf, Germany

## Abstract

**Background:**

Higher plants evolved various strategies to adapt to chilling conditions. Among other transcriptional and metabolic responses to cold temperatures plants accumulate a range of solutes including sugars. The accumulation of the reducing sugars glucose and fructose in mature potato tubers during exposure to cold temperatures is referred to as cold induced sweetening (CIS). The molecular basis of CIS in potato tubers is of interest not only in basic research on plant adaptation to environmental stress but also in applied research, since high amounts of reducing sugars affect negatively the quality of processed food products such as potato chips. CIS-tolerance varies considerably among potato cultivars. Our objective was to identify by an unbiased approach genes and cellular processes influencing natural variation of tuber sugar content before and during cold storage in potato cultivars used in breeding programs. We compared by two-dimensional polyacrylamide gel electrophoresis the tuber proteomes of cultivars highly diverse for CIS. DNA polymorphisms in genomic sequences encoding differentially expressed proteins were tested for association with tuber starch content, starch yield and processing quality.

**Results:**

Pronounced natural variation of CIS was detected in tubers of a population of 40 tetraploid potato cultivars. Significant differences in protein expression were detected between CIS-tolerant and CIS-sensitive cultivars before the onset as well as during cold storage. Identifiable differential proteins corresponded to protease inhibitors, patatins, heat shock proteins, lipoxygenase, phospholipase A1 and leucine aminopeptidase (Lap). Association mapping based on single nucleotide polymorphisms supported a role of Lap in the natural variation of the quantitative traits tuber starch and sugar content.

**Conclusions:**

The combination of comparative proteomics and association genetics led to the discovery of novel candidate genes for influencing the natural variation of quantitative traits in potato tubers. One such gene was a leucine aminopeptidase not considered so far to play a role in starch sugar interconversion. Novel SNP’s diagnostic for increased tuber starch content, starch yield and chip quality were identified, which are useful for selecting improved potato processing cultivars.

## Background

Sessile higher plants evolved various strategies to adapt to chilling conditions. Among other transcriptional and metabolic responses to cold temperatures plants accumulate a range of solutes including amino acids, glucosides or sugars. Although the precise function of sugars remains to be elucidated, their accumulation suggests roles as osmoregulators, cryoprotectants or signaling molecules. In mature potato tubers, the accumulation of soluble sugars during cold adaptation is referred to as cold induced sweetening (CIS) [1-3]. The sugars sucrose, glucose and fructose accumulating in photosynthetic inactive tissues like potato tubers are recruited from starch degradation [3,4]. Enzymes involved in starch and sugar metabolism have been identified and studied intensively in potato and other plants at the biochemical and molecular level [5-7]. However, the regulation of this process is not entirely understood. Various enzymes, such as amylase, UDP glucose pyrophosphorylase or invertase, have been suggested to control the level of CIS in tubers either by increased or suppressed expression or activity [8-10]. The activity of invertase, which converts sucrose into glucose and fructose, is apparently subject to post-translational regulation by proteinaceous inhibitors [11-15].

Beside functions in plant adaption to cold temperatures CIS is an important issue for the potato processing industry. Long term storage of potato tubers at low temperatures is advantageous to reduce sprouting, thereby extending marketability. However, high concentrations of the reducing sugars glucose and fructose, either inherently or caused by CIS, negatively affect the quality of processed food products such as potato chips and French fries [16].

Potato cultivars show extensive natural variation in CIS capacity [17,18]. Diversity in tuber sugar content might be explained by the variation of abundance and/or activity of carbohydrate metabolizing enzymes in source (photosynthetic leaves) and sink tissues (tubers), and by variable flux of sucrose from source to sink. Knowledge of the molecular basis of the diversity will contribute to the deciphering of plant cold adaptation and the development of diagnostic markers that can be used to select cultivars with low capacity of sugar accumulation and therefore improved processing quality.

Tuber starch and sugar content are quantitative traits controlled by multiple genetic and environmental factors. Molecular linkage mapping of quantitative trait loci (QTL) and candidate genes revealed co-localization of some QTL for tuber starch and sugar content with genes functional in carbohydrate metabolism or transport [17,19-21]. More recently, association genetics demonstrated that DNA polymorphisms in genes encoding invertases and starch phosphorylases were associated with potato chip color, starch content and starch yield [22-24]. The genetic analyses support the working model that natural variation in tuber starch and sugar content is controlled by allelic variants of enzymes that function in starch and sugar metabolism. However, this model explains only part of the observed genetic variation.

To identify novel factors influencing sugar accumulation in tubers, unbiased and comprehensive approaches such as transcriptome and proteome profiling are required. Microarray hybridization experiments using a tomato gene chip hybridized with potato RNA allowed the identification of known as well as novel genes that were differentially expressed during tuber cold storage in a single potato genotype. Transcript levels of known candidate genes, such as invertase, were correlated with sugar accumulation [25]. Analyzing the proteome captures both the variation of transcript abundance and the variation of post-transcriptional processes in biological samples, and differential expression of proteins reflects the translated genome in any environment or stress [26]. Comparative proteome analysis has previously proven successful in identifying new candidate genes for controlling tuber quality traits [27-29].

The objective of this study was to decipher the molecular basis of natural variation in tuber reducing sugar content (RSC) and cold induced sweetening (CIS) ability in elite breeding materials of *Solanum tuberosum*. Using 2D-PAGE (two-dimensional polyacrylamide gel electrophoresis) we compared the tuber proteome of forty potato cultivars showing high diversity in tuber reducing sugar content and detected correlations between the expression level of distinct proteins and RSC. In addition a cold storage experiment was performed to tackle proteins differentially expressed in response to cold treatment as well as between CIS-tolerant (CIS-t) and CIS-sensitive (CIS-s) cultivars. Comparative 2D-PAGE protein profiling of pools of CIS-tolerant and CIS-sensitive genotypes at certain time points during cold storage identified at least fifty differentially expressed proteins. SNPs at two selected candidate loci encoding proteins either correlated with RSC or differentially expressed between CIS-tolerant and CIS-sensitive cultivars throughout cold storage were tested for association with tuber starch and chip quality in order to validate their potential role in controlling natural variation of these traits. Our approach led to the discovery of novel, potential regulatory genes that have not been connected so far to plant carbohydrate metabolism.

## Results

### Tuber reducing sugar content (RSC) before and after cold treatment

**Figure 1:**
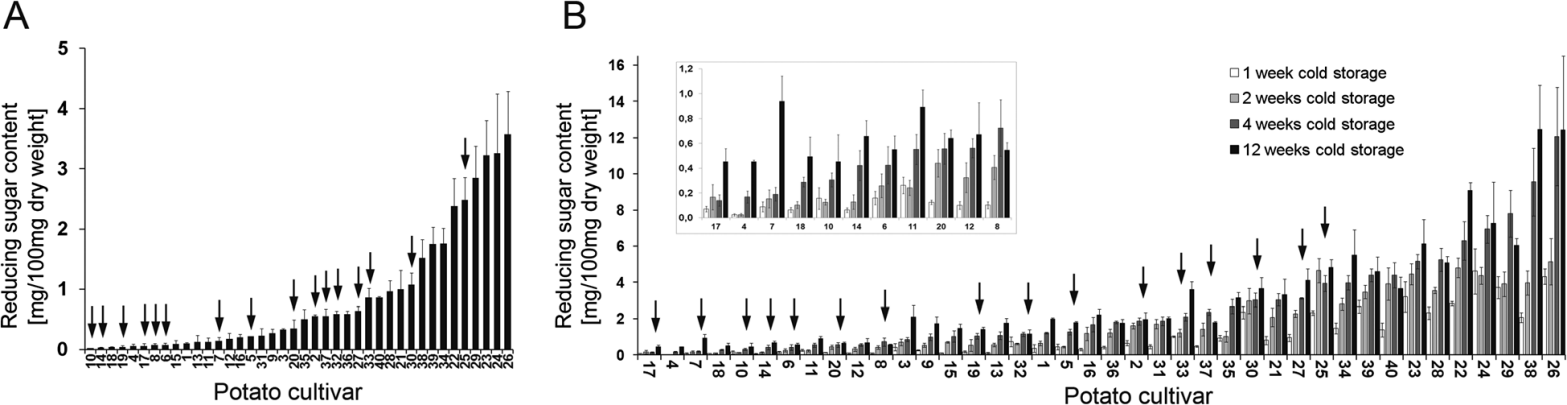
**Reducing sugar content (RSC) in tubers of 40 cultivars during cold storage.** RSC represents the amounts of glucose and fructose in freeze dried tuber tissue. Cultivars are coded by numbers shown in Additional file 1: Table S1. **(A)** RSC before cold storage. Cultivars are ranked according to increasing RSC. **(B)** RSC after 1, 2, 4 and 12 weeks of cold storage. Cultivars are ranked according to increasing RSC after 4 weeks of cold storage. The arrows indicate cultivars that contain at least one dosage of a leucine aminopeptidase allele, for which the SNP allele *A*_*2746*_ is characteristic. SNP*2746* associated highly significant with tuber starch content, starch yield and chip quality after storage at 4°C (Table 3). The inset presents a magnification of the data obtained for cultivar 17 to cultivar 8.

Tuber RSC was determined before and after cold treatment in 40 cultivars pre-selected for high (No.1 to 20, Additional file 1: Table S1) and low (No. 21 to 40, Additional file 1: Table S1) chip quality. Before cold storage, RSC varied from 0.02 to 3.58 percent dry weight (Figure 1A). The ranking of cultivars corresponded to their prior allocation based on chip quality with two exceptions (cultivars 2 and 31). During twelve weeks of storage at 4°C, RSC increased in all cultivars (Figure 1B). Cultivars exhibiting low RSC values before cold storage showed a higher increase in RSC during cold storage compared to cultivars with high initial RSC values. However, the ranking of cultivars according to RSC before and after 4 weeks cold storage was in high agreement with their assignment to high and low chip quality. In conclusion, RSC was highly indicative for chip quality and vice versa.

### Correlation between RSC and protein spot intensity without cold treatment

**Figure 2:**
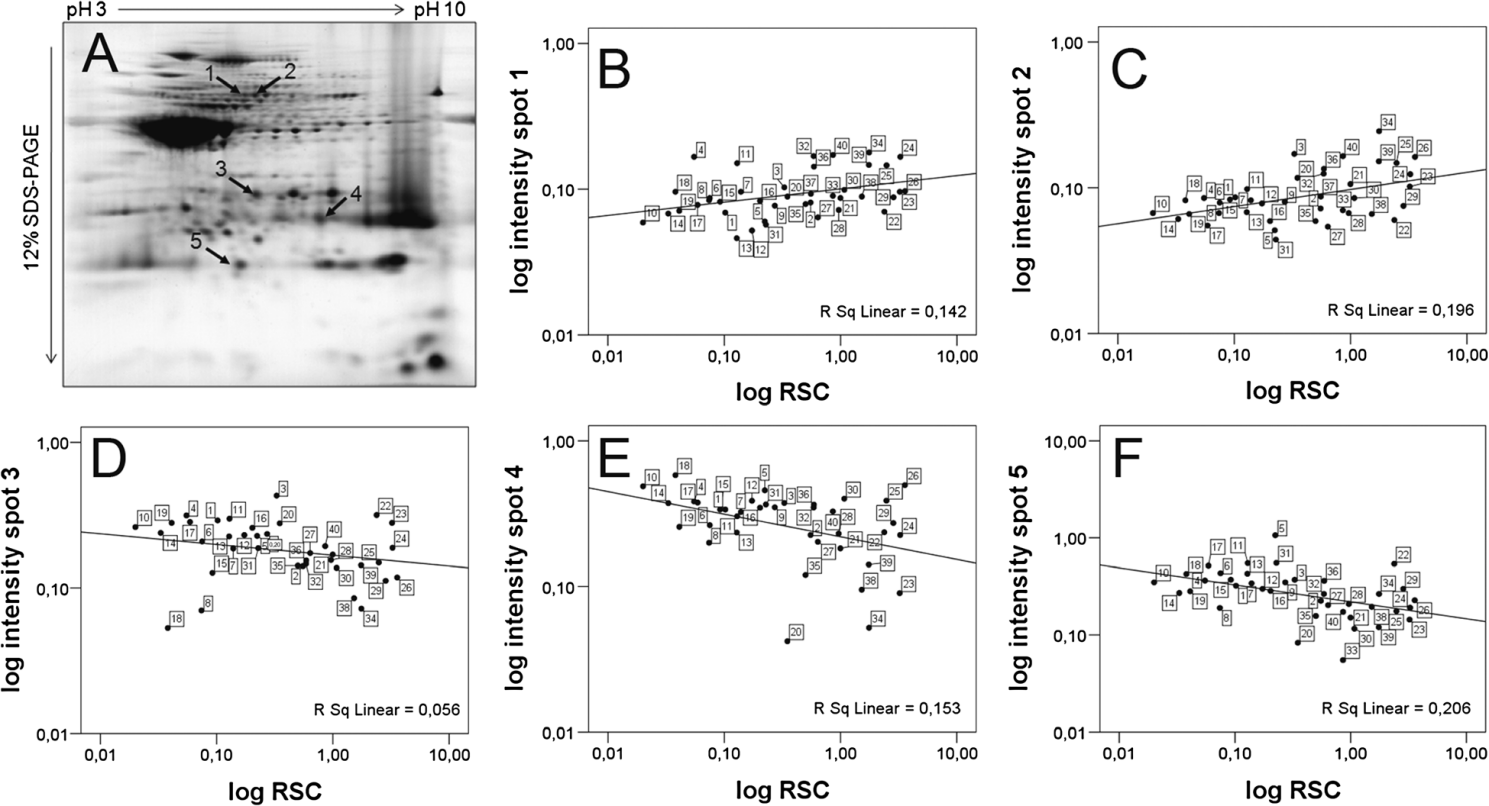
**Correlation between mean protein spot intensity and reducing sugar content (RSC) in 40 cultivars without cold treatment. (A)** Representative pattern of tuber proteins obtained by 2D-PAGE. Numbered arrows point to the five spots with significant correlation between mean spot intensity and RSC (Table 1). **(B-F)** Correlation plots of the logarithmic values of mean spot intensity versus RSC. Plots **B**, **C**, **D**, **E** and **F** correspond to spots 1, 2, 3, 4 and 5, respectively. Framed numbers indicate potato cultivars listed in Additional file 1: Table S1.

Total soluble protein of tubers of the 40 cultivars before cold treatment was separated by 2D-PAGE. An average of 226 protein spots was detected per gel (data not shown). Twenty six protein spots were selected based on being present in more than 70% of all gels of the 40 cultivars including biological replicates, and quantified by measuring spot intensity. The data for mean RSC and spot intensities are provided in Additional file 2: Table S2. The spot intensity of five proteins correlated significantly (p < 0.05) with RSC (Figure 2, Table 1). The most significant correlation was found for a protein identified as ‘putative Kunitz-type tuber invertase inhibitor’ (Figure 2F, Table 1, Additional file 3: Table S3). Decreasing protein spot intensity correlated with increasing RSC. Negative correlation coefficients were also obtained for two proteins identified as ‘miraculin’ and ‘potato proteinase inhibitor class II’ (Figure 2D and E, Table 1, Additional file 3: Table S3). Two further proteins spots, both identified as ‘granule bound starch synthase’ were positively correlated with RSC (Figure 2B and C, Table 1, Additional file 3: Table S3).

**Table 1:** **Proteins showing significant (**ρ **< 0.05) correlation between mean spot intensity and tuber sugar content in 40 cultivars**

**Spot No**	**PPMCC **^**1**^	**ρ-value**	**Protein identity**	**Accession**	**Locus **^**2 **^**PGSC0003DMG**	**Superscaffold PGSC0003DMB**	**Chromosome**
1	0,428	0,006	Granule-bound starch synthase	1718316A	400012111	000000048	VIII
2	0,403	0,010	Granule-bound starch synthase	1718316A	400012111	000000048	VIII
3	-0,345	0,029	Miraculin; Precursor	P13087	- ^3^	- ^3^	?
4	-0,518	0,001	Putative Kunitz-type tuber invertase inhibitor precursor	AAL60242	400010146, 40001043, 400010139	000000159	III
5	-0,355	0,025	Proteinase inhibitor II	AAO88244	400004547 400004548	000000400	III

^1^Pearson product–moment correlation coefficient.

^2^When proteins matched to multiple loci in the potato genome, they were ordered according to decreasing sequence similarity.

^3^No sequence similarity was detected.

### Comparative protein profiling during cold treatment

**Figure 3:**
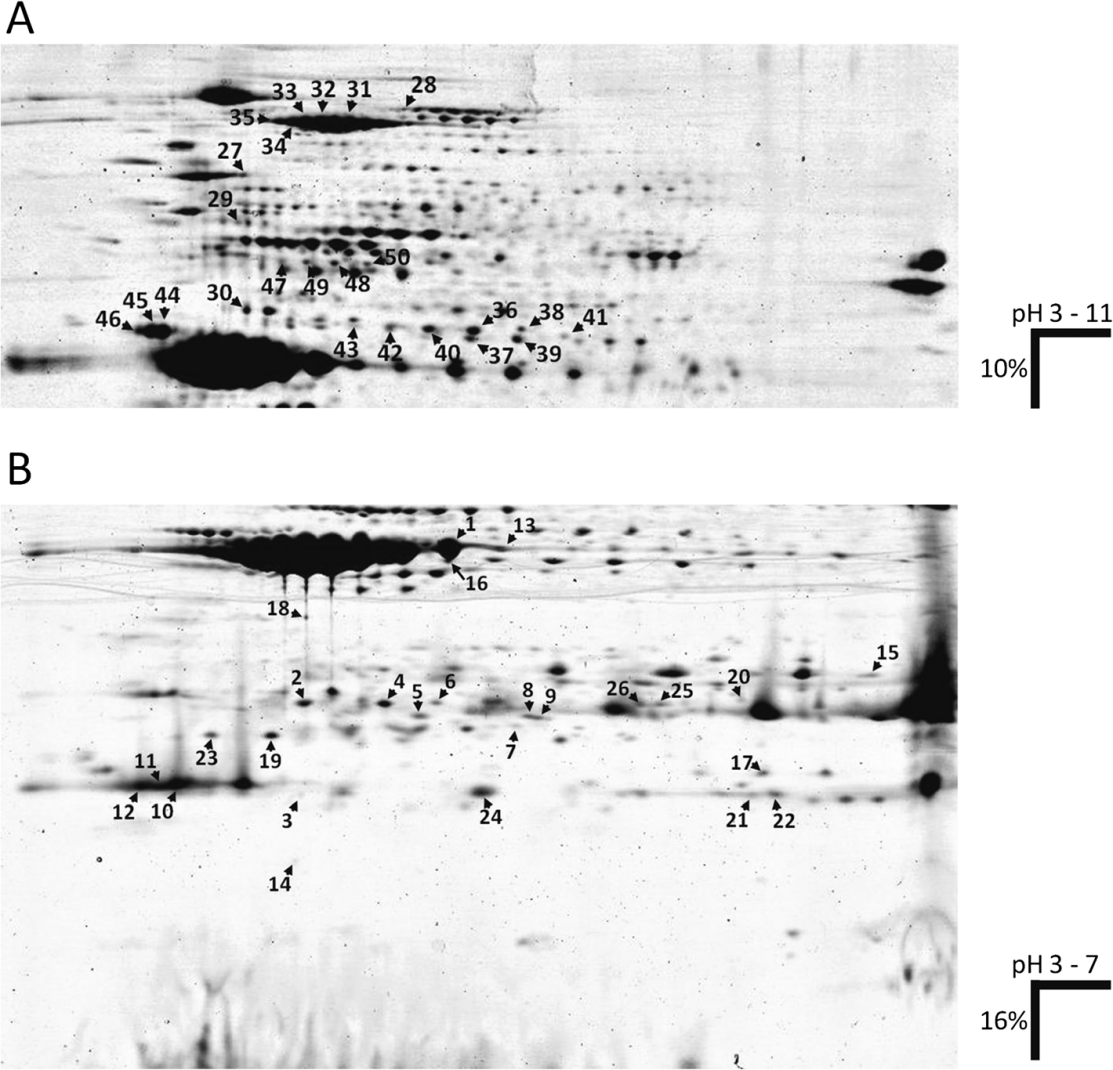
**Protein spots showing differential expression between genotype pools CIS-t** and **CIS-s before and/or after cold treatment.** A virtual tuber protein pattern was generated by fusing 2D-PAGE gel images from both genotype pools **CIS-t** and **CIS-s** at T0. Conditions for protein separation in the first and second dimension are shown on the right. The numbered arrow heads point to the position of the 50 differential proteins described in Table 2, Additional file 4: Table S4 and Additional file 5: Table S5. **(A)** Proteins from 40 to 200 kDa were separated on IPG strips with immobilized pH gradient of 3–11 in the first dimension (IEF) and by 10% Tris-glycine SDS-PAGE in the second dimension. **(B)** Proteins between 5 and 40 kDa were separated on pH 3–7 IPG strips in the first dimension and by 16% Tris-tricine polyacrylamide gels in the second dimension.

Based on the accumulation of reducing sugars during cold treatment (Figure 1B) 10 cultivars were selected, five each with the lowest (CIS-tolerant, CIS-t) and highest (CIS-sensitive, CIS-s) RSC values (indicated in Additional file 1: Table S1). Total soluble protein was extracted from tubers of the CIS-t and CIS-s cultivars prior to (T0) and after 2, 4 and 12 weeks of cold treatment (2w, 4w, 12w). At each time point two protein pools were created. The first pool was made of equimolar amounts of proteins from CIS-t cultivars and the second from CIS-s cultivars. The protein pools were separated by 2D-PAGE under two different conditions to resolve proteins of different size classes (Figure 3). By comparing protein profiles of the genotype pools CIS-t and CIS-s, protein spots were identified that showed quantitative as well as qualitative differences at one or more time points. Fifty protein spots (Figure 3) showed a minimum of a two-fold difference in spot intensity between genotype pools CIS-t and CIS-s at least at one time point (Table 2). The kinetics of the spot intensities during cold treatment and genomic positions of the corresponding genes are shown in Additional file 4: Table S4.

**Table 2:** Tuber proteins differentially expressed in genotype pool CIS-t versus pool CIS-s during 12 weeks storage at 4°C

**Spot**	**Protein**	**Accession no.**	**Ratio of mean spot volume**^**1**^			
			**T0**	**2w**	**4w**	**12w**
**Protease inhibitors**						
2	Serine protease inhibitor 7	P30941.2	0,24 **	0,19 **	0,28 **	0,28 **
4	Serine protease inhibitor 7	P30941.2	0,28 **	0,23 **	0,22 *	0,16 *
3	Kunitz-type enzyme inhibitor S9C11	AAL67830.1	0,37 *	0,43 **	0,49 **	0,6 *
6	Putative miraculin	CAC40756.1	0,58 *	0,53 **	0,62 **	0,42 *
14	Aspartic protease inhibitor 5	P58519.1	0,69	0,37 *	n.d.	0,65 *
10	Kunitz-type proteinase inhibitor	AAM21645.1	0,77	0,44 **	0,71 *	0,23 *
11	Kunitz-type proteinase inhibitor	AAM21645.1	0,74	n.d.	0,24 ***	n.d.
12	Kunitz-type proteinase inhibitor	AAM21645.1	0,48	0,51 *	0,52 **	0,43 **
19	Putative miraculin	CAC40756.1	3,64 *	5,02 **	2,85 **	4,34 **
20	Cysteine protease inhibitor 1	P20347.3	n.d.	4,21 ***	2,81 ***	3,32 **
21	Proteinase inhibitor II	CAA27730	1,54	2,89 **	1,62 *	0,99
22	Proteinase inhibitor II	CAA27730	1,48	2,09 *	1,45 *	1,59 **
24	Proteinase inhibitor II	CAA27730	2,89 *	1,56 *	1,25 *	1,59 *
**Storage proteins**						
1	Patatin-2-Kuras 4	Q3YJT0	0,43 *	0,39 *	0,55 ***	0,47 **
13	Patatin Group M-3	Q2MY51	0,24	0,38 **	0,63 *	0,40 *
44	Patatin-3-Kuras 1	Q3YJS9.1	2,57 *	3,37 *	3,22 *	2,04
45	Patatin-3-Kuras 1	Q3YJS9.1	2,99 *	2,67 *	3,20 *	2,60 *
46	Patatin-3-Kuras 1	Q3YJS9.1	1,51	2,26	2,07 *	1,97
**Lipid metabolism**						
31	Lipoxygenase	X95512.1	1,42 **	1,56 **	1,94 **	2,17 **
32	Lipoxygenase	X95512.1	1,56 *	2,02 **	1,65 **	1,79 *
33	Lipoxygenase	X95512.1	1,52	2,78 **	1,73 **	1,59 *
34	Lipoxygenase	X95512.1	1,66	3,71 *	1,72 **	1,51 *
35	Lipoxygenase	X95512.1	1,45	3,90 *	1,71 *	1,65
36	Phospholipase A1	ABQ95989.1	3,20 ***	2,82 ***	7,45 **	2,92 *
39	Phospholipase A1	ABQ95989.1	4,22 ***	2,59 ***	3,52 **	2,76 **
40	Phospholipase A1	ABQ95989.1	3,65 **	2,57 **	1,59 **	2,27 *
**Glycolysis**						
43	Phosphoglycerate kinase	ABB87110	0,79	2,29 *	n.d.	n.d.
**Heat shock proteins**						
5	Chloroplast small heat shock protein class I	AAQ19680.1	0,24 **	0,23 **	0,25 *	0,27 *
27	Heat shock protein (HSP70)	XP_002512741	0,87	0,40 *	n.d.	0,68 *
28	101 kDa heat shock protein	AAC83688.2	0,85	1,13	0,48 **	0,98
**Proteases**						
47	Leucine aminopeptidase, chloroplastic	P31427.2	2,35 *	2,16 **	1,47 *	n.d.
48	Leucine aminopeptidase, chloroplastic	P31427.2	1,82 *	1,19	3,06 **	2,16 *
49	Leucine aminopeptidase, chloroplastic	P31427.2	1,43	1,78 **	2,22 **	1,66
50	Leucine aminopeptidase, chloroplastic	P31427.2	n.d.	n.d.	2,16 **	1,26
**Pathogenesis related proteins**						
17	Putative PR-10 type pathogenesis-related protein	BAJ25784.1	0,39 *	0,75	0,93	0,79
**Cytoskeleton**						
30	Actin	BAK57343.1	0,47 **	1,06	0,72 *	1,20
**Unidentified proteins**						
7	Not identified		0,40 **	0,57 **	0,57 **	0,52 **
8	Not identified		0,60 *	0,44 *	0,39 *	0,22 *
9	Not identified		0,26**	0,14**	n.d.	n.d.
15	Not identified		0,46	0,46 *	0,29 *	0,44 *
16	Not identified		n.d.	0,12 ***	0,09 ***	0,26 ***
18	Not identified		3,6 **	1,73 *	1,63*	1,45
23	Not identified		3,64 *	1,62	2,04 *	3,12 **
25	Not identified		2,20 **	n.d.	2,80 **	n.d.
26	Not identified		1,72	n.d.	2,64 ***	2,69 *
29	Not identified		0,99	0,73 *	0,77	0,48 **
37	Not identified		3,96 **	4,25 **	4,90 **	3,96 **
38	Not identified		3,06 *	2,85 ***	3,88 ***	2,15
41	Not identified		2,52	1,62 *	2,06 **	2,54 *
42	Not identified		1,68 *	2,99 **	2,78 **	n.d.

^1^Ratio of the mean spot volume of the CIS-t pool to the mean spot volume of the CIS-s pool. Mean spot volume was calculated from three biological replicates. The level of significance is indicated by * for 0.05 > p >0.01, ** for 0.01 > p >0.001 and *** for p <0.001.

n.d. spot not detectable in 2D protein profiles.

2w, 4w and 12w corresponds to weeks of storage at 4°C.

Thirty six of fifty differential protein spots were assigned to various protease inhibitors (PI’s), isoforms of the storage protein patatin, a lipoxygenase (Lox), a phospholipase A1 (PLA1), phosphoglycerate kinase (PGK), several heat shock proteins (HSP), a leucine aminopeptidase (Lap), pathogenesis related protein 10 (PR10) and an actin (Data on protein identification provided in Additional file 5: Table S5). The largest group corresponded to protease inhibitors.

Twenty eight protein spots were differentially expressed before the onset of cold treatment, seventeen at higher level in genotype pool CIS-t and eleven in pool CIS-s. For example, three series of spots identified as Lox (spots 31–35, Figure 3A), PLA1 (spots 36, 39 and 40) and Lap (spots 47–50) showed higher expression in pool CIS-t compared to pool CIS-s. In contrast, Kunitz-type protease inhibitors (spots 2, 3, 4 and 6) and HSP’s (spot 5) were more abundant in pool CIS-s versus CIS-t.

Significant differential expression of eighteen proteins was maintained at all time-points during cold treatment. Of major interest are spots 6 and 19, which migrated at different pI and molecular weight but were assigned to the same miraculin accession when searching the NCBI protein database. The tryptic peptides matched to different loci on chromosomes III and XII in the potato genome sequence [30]. The miraculin gene on chromosome III was consistently higher expressed in pool CIS-t and the miraculin gene on chromosome XII in pool CIS-s (Additional file 4: Table S4).

Additional expression patterns observed were: induction by cold treatment either in genotype pool CIS-t (e.g. spot 20) or CIS-s (e.g. spots 11, 14) or in both pools (e.g. spots 3, 7, 16), either transiently (e.g. spots 10, 11, 50) or continuously (e.g. spots 7, 20), or repression by cold treatment, either in pool CIS-t (e.g. spots 24, 38) or CIS-s (e.g. spot 17) or in both pools (e.g. spot 25).

### Genomic organization of candidate genes and association with tuber quality traits

Single nucleotide polymorphisms (SNP’s) at two loci encoding the differentially expressed proteins ‘putative Kunitz-type tuber invertase inhibitor’ (AAL60242) and ‘chloroplastic leucine aminopeptidase’ (P31427) were tested for association with tuber quality traits in the CHIPS-ALL association mapping population [22].

### a) Putative Kunitz-type tuber invertase inhibitor (KT-InvInh)

BLAST analysis of the corresponding nucleotide sequence (AF459077) against the potato genome sequence [30] and the GeneBank nucleotide core collection revealed 99.7% sequence identity with the locus PGSC0003DMG400010146 on superscaffold PGSC0003DMB000000159 (chr03:19743564..21131995) and 99% sequence identity with potato BAC clones BA259D20 and BC135N2 [31]. This positioned *KT-InvInh* within a mixed cluster of protease inhibitor gene families at the *StKI* locus on potato chromosome III [30, 31, 32]. Locus specific primers were designed based on unique sequences flanking gene PGSC0003DMG400010146, which were used to generate and sequence an amplicon from the individuals of the CHIPS-ALL population. Fourteen SNPs and one insertion/deletion (indel) polymorphism (Additional file 6: Figure S1) were evaluated for association with tuber starch content (TSC), tuber yield (TY), tuber starch yield (TSY), chip quality after harvest in autumn (CQA) and after cold storage (CQS) (Table 3). Nine SNPs and the indel were associated with TSC and TSY. The SNPs fell into three haplotype groups with highly similar distribution in the CHIPS-ALL population and therefore similar associations. All minor frequency SNP alleles increased average TSC and TSY (Table 3). Few small associations (0.05 > p > 0.01) were found with TY (not shown).

**Table 3:** SNPs/Indels associated with tuber starch content (TSC), starch yield (TSY), chip quality after harvest (without cold storage, CQA) and 3 months cold storage (CQS) in the CHIPS-ALL population

**Gene**	**Alleles **^**1**^	**Minor allele frequency**	**CQA **^**2 **^***R***^***2 ***^**[%]**	**CQS *R***^***2 ***^**[%]**	**TSC *R***^***2 ***^**[%]**	**TSY *R***^***2 ***^**[%]**
*Putative Kunitz-type tuber invertase inhibitor (KT-InvInh)*	A/*G*_261_ (±*GATGAT*_114_, G/*T*_334_)	0.20 (*G*_261_)	ns	ns	11.2*** ↑	11.6*** ↑
	C/*G*_206_ (C/*G*_352_, C/*G*_362_, A/*T*_391_, A/G/*C*_430_)	0.13 (*G*_206_)	ns	ns	6.5** ↑	8.7*** ↑
	A/*G*_395_ (G/*A*_396_)	0.02 (*G*_395_)	ns	ns	7.2*** ↑	9.4*** ↑
*Leucine aminopeptidase, neutral (LapN)*	G/*A*_2746_	0.11 (*A*_2746_)	4.1** ↑	10.5*** ↑	17.5*** ↑	14.1*** ↑
	T/*C*_3117_	0.16 (*C*_3117_)	ns	4.3* ↑	7.4** ↑	6.1** ↑

^1^Alleles with highly similar distribution in the CHIPS-ALL population and similar associations are shown in parentheses. SNP alleles forming the minor frequency haplotype are italics.

^2^ns: not significant. The level of significance is indicated by * for 0.05 > p >0.01, ** for 0.01 > p >0.001 and *** for p <0.001. Arrows indicate the direction of effect of the minor frequency allele, which increased average tuber starch content, starch yield and chip quality in all cases.

### b) Chloroplastic leucine aminopeptidase (Lap)

**Figure 4:**
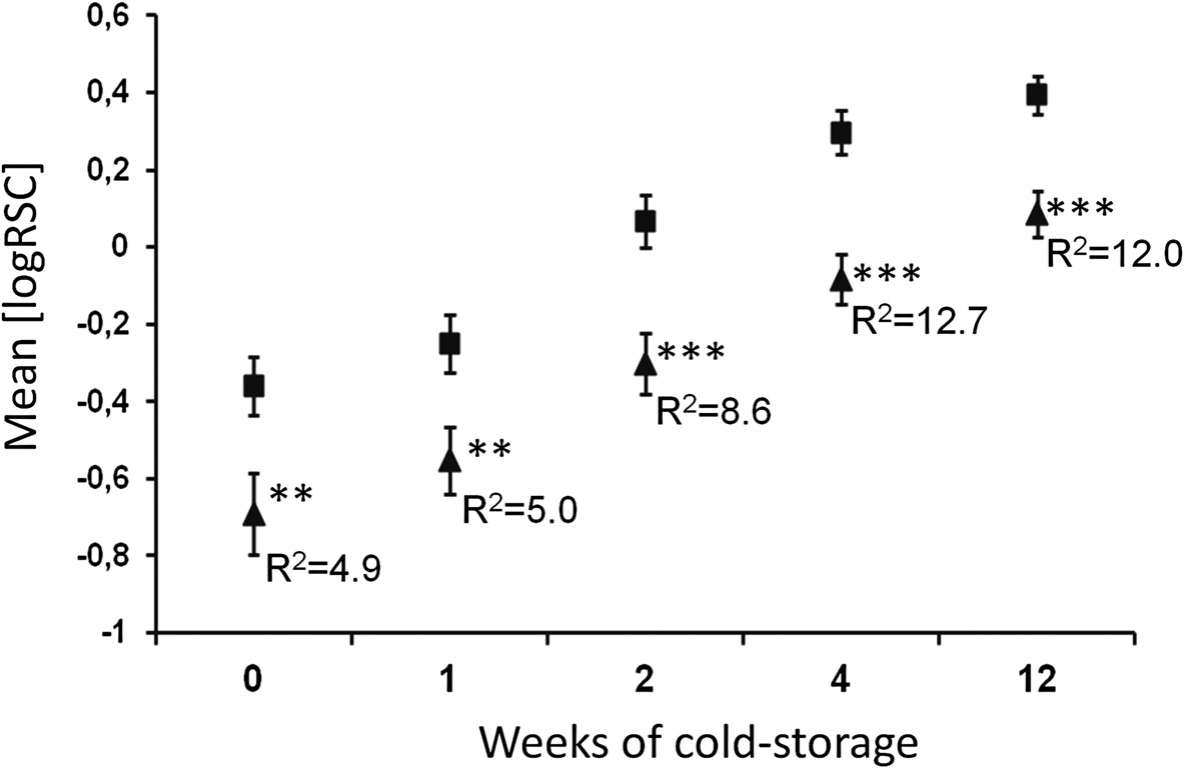
**Effect of the SNP allele *StLapN-A***_***2746***_**on average RSC of 40 cultivars during cold storage.** Cultivars were grouped according to presence (triangles) or absence (squares) of SNP allele A_2746_. The genotypic groups were tested by ANOVA for significant differences between means of RSC (log transformed) before and after 1, 2, 4 and 12 weeks of cold storage. The mean RSC was different between the genotypic groups at all time points (**: 0.01 > p <0,001; ***: p < 0.001). The amount of variance explained by *StLapN-SNP*_*2746*_ (R^2^) at the different time points is given as percentage. R^2^ values increased during cold storage.

BLAST searches with the cDNA sequence X77015 corresponding to the differential Lap protein against the potato genome sequence [30] identified superscaffold PGSC0003DMB000000116 (chr12:910581..2552409), which contained a single gene model annotated as ‘neutral Lap’ (*StLapN*). The same superscaffold contained the marker loci *AGPaseB-b* (PGSC0003DMG400046891) and *GP34*[32], which anchored the superscaffold to the distal part of the long arm of potato chromosome XII. The sequence X77015 clearly matched to two regions (> 95% sequence identity), indicating a tandem repeat of two *Lap* genes with ten exons each, located within a 17 kbp region of superscaffold PGSC0003DMB000000116 (chr12:910581..2552409). The genomic organization was confirmed by aligning tomato *Lap* sequences U50151 (acidic *Lap*, *SlLapA*) and AF510743 (neutral Lap, *SlLapN*) with the superscaffold. The cDNA sequence of accession X77015 was more similar to *SlLapA* (94.6%) than to *SlLapN* (86.2%) and *StLapN* (87.2%), indicating that the differential protein annotated as ‘chloroplastic leucine aminopeptidase’ corresponded to *StLapA*. A specific amplicon suitable for sequencing and SNP detection could only be obtained from the physically closely linked *StLapN* gene. Seventeen SNPs and one indel (Additional file 6: Figure S1) were tested for association with the tuber traits in the CHIPS-ALL population. Four SNPs were associated with one or more tuber trait, two of which showed only very small effects (data not shown). SNP_2746_ showed highly significant associations with tuber starch content (TSC), tuber starch yield (TSY), chip quality after harvest (CQA) and chip quality after cold storage (CQS). Cultivars containing the minor frequency allele A_2746_ showed significant increased average trait values (Table 3). Furthermore, when scored in the 40 cultivars evaluated for RSC during cold storage, the SNP allele A_2746_ was detected in 16 cultivars, preferentially in cultivars with low RSC and particularly after four weeks cold storage (Figure 1). This observation was confirmed when the 40 cultivars were grouped based on presence and absence of the SNP allele A_2746_ and tested for difference between the RSC group means. At all time-points, the genotypic group having at least one dosage of the A_2746_ allele accumulated significantly lower amounts of reducing sugars than the group homozygous for the allele G_2746_ (Figure 4). The positive effect of SNP_2746_ increased during cold storage, consistent with the result obtained in the CHIPS-ALL population.

## Discussion

Comparative proteomics in combination with association genetics was used to decipher the molecular basis of natural variation in reducing sugar accumulation and CIS-tolerance in potato tubers. We identified proteins, the abundance of which correlated with tuber sugar content. Moreover, we identified proteins showing differential expression between groups of cultivars with high and low CIS-tolerance before and during cold storage.

Differential protein levels may be the cause or the consequence of the process leading to a phenotype such as tuber sugar content. The genes controlling phenotypic variation are a subset of all genes that have a metabolic, structural or regulatory function in phenotypic expression. Accordingly, DNA polymorphisms should be present in coding or regulatory regions of causal genes that are associated with the phenotypic variation. Comparative protein profiling and association genetics are therefore complementary approaches for zooming in on genes controlling complex traits. DNA polymorphisms at two loci encoding the differential proteins KT-InvInh and StLapA were associated with tuber quality traits like tuber starch content, tuber starch yield or chip quality. Consequently, they represent good candidate genes for contributing to the natural variation in tuber RSC and CIS tolerance. To be useful for breeding applications, the genetic material used for association analysis should originate from state of the art breeding programs. Our association panel, which consisted of varieties and advanced clones of commercial breeding programs in central Europe fulfilled this criterion [22]. The newly discovered SNPs associated with chip quality, tuber starch content and starch yield in this genetic material can be used for marker assisted selection of superior processing cultivars.

For the identification of proteins up or down regulated in response to cold treatment and influencing natural variation in CIS tolerance we compared tuber protein profiles of CIS-s and CIS-t genotype pools during cold storage. The majority of the differential protein spots (28 out of 50) showed significant differences between genotype pools CIS-t and CIS-s before the onset of cold treatment (Table 2, Additional file 4: Table S4). These differences were generally maintained throughout cold storage. Consequently, our results suggest that these constitutive differences in protein expression are involved in that part of RSC, which is independent from cold treatment.

Among the differential spots were novel proteins such as leucine aminopeptidase that has not been considered so far to play a role in starch-sugar interconversion. Others like differential heat shock proteins corroborated previous findings [25,28]. In contrast, several genes known to be associated with tuber sugar and starch content [22] were not detected as differentially expressed proteins, most likely due to limited resolution and sensitivity of 2D-PAGE. Nevertheless, this study demonstrates that comparative proteomics is suitable to identify novel candidate genes and yields results complementary to association mapping based on functional and positional candidate genes.

### Proteins correlated with tuber sugar content

The most significant correlation was found for a putative Kunitz-type invertase inhibitor (KT-InvInh). This protein was previously purified from the soluble tuber protein fraction and was shown to inhibit soluble tuber invertase *in vitro*[15]. The role of invertase in cold sweetening of potato tubers has been characterized previously [33-36]. Invertase enzymatic activity increased during cold storage, whereas an invertase inhibitory activity decreased, suggesting that invertase activity and thereby reducing sugar accumulation are regulated by a specific invertase inhibitor [14,33,37]. The negative correlation of KT-InvInh protein abundance with tuber sugar content is consistent with this model. KT-InvInh has no sequence similarity with other cloned invertase inhibitors of tobacco and potato [11-13,38,39].

Association analysis of *KT-InvInh* identified highly significant associations of nine SNPs and one indel with tuber starch content and starch yield but not with chip quality (Table 3). Interestingly, SNP_307_ (Additional file 6: Figure S1) was not associated with any trait. SNP_307_ causes the change from Valine (G_307_) to Methionine (A_307_), which was characteristic for the KT-InvInh protein [15]. The allele *A*_*307*_ had a frequency of 93% in the CHIPS-ALL population and was therefore nearly fixed. This might explain the lack of association with chip quality. The associations observed at the *KT-InvInh* locus might also result from linkage disequilibrium with other differentially expressed Kunitz-type inhibitors that are physically linked to *KT-InvInh* (see Additional file 4: Table S4 and below). Alternatively, KT-InvInh may target *in vivo* a protein, which regulates starch degradation. In this case KT-InvInh allelic variants could have a causal effect on tuber starch content and starch yield.

The two additional inhibitors negatively correlated with RSC were a miraculin and a potato inhibitor 2 (PIN2). Similarly to miraculins, PIN2s have not been considered as candidate genes in the context of starch-sugar interconversion. As correlations between two variables do not provide conclusive evidence for a causal relationship between them, the effect of protease inhibitors on sugar accumulation may be direct or indirect.

### Proteins differentially expressed between genotype pools CIS-t and CIS-s

Fifty protein spots differed qualitatively or quantitatively between ten genotypes accumulating either low (CIS-t) or high reducing sugar levels (CIS-s) before and during cold treatment. Due to high sequence similarity between the members of some gene families, an unambiguous locus could not be assigned to all protein spots. Different spots matching to the same protein accession can be allelic variants of the same gene. Alternatively, spots derived from different genes can match to the same protein accession due to limited resolution of the protein databases. Among the annotated spots were isoforms of the most abundant tuber proteins patatin and protease inhibitors (PI) [27,40-42]. Thirteen differentially expressed proteins were Kunitz-type protease inhibitors (KPI) or potato inhibitors 2 (PIN2). KPIs and PIN2s are encoded by multi-gene families organized in mixed clusters at the *StKI* locus on potato chromosome III [31,43]. Several studies have demonstrated high sequence variability and genotype specific variants among potato protease inhibitors [29,41-45]. The structural variation may indicate functional variation. Apart from proteases, enzymes like invertase and amylase were inhibited by PIs [15,42,46-48]. The genotype dependent, and in some cases cold induced or repressed expression suggests a regulatory role of several PIs in starch-sugar interconversion.

Remarkable differences between CIS-t and CIS-s genotypes were detected for patatin and phospholipase A1 (PLA1) isoforms. The role of patatin in potato tubers has been described as storage proteins that serve as nitrogen source for sprouting and early plant growth. The *in vitro* lipid acyl hydrolase (LAH) and phospholipase activity of patatin and related proteins of other plants suggest additional functions [49-51]. Phospholipases are involved in phospholipid and galactolipid degradation leading to changes in membrane composition and properties during cold acclimation [52,53]. Increased membrane fluidity will lower the threshold temperature for membrane damage and will maintain activity of membrane associated proteins. Such a role of patatins and phospholipase A1 in altering membrane lipid composition could explain differences between genotypes with strong (CIS-s) and weak (CIS-t) cold acclimation responses.

Lipoxygenase (Lox) is a dioxygenase which catalyses the hydroperoxidation of polyunsaturated fatty acids. The Lox that was differentially expressed between genotype pools CIS-t and CIS-s corresponded to a 9-Lox. 9-Lox oxidizes preferentially linoleic acid to 9(S)-hydroperoxy linoleic acid, which is the predominant hydroperoxide isoform in potato tubers [54,55]. Metabolites generated by 9-Lox were involved in tuber development. Suppression of 9-Lox activity in tubers correlated with reduced tuber yield, decreased tuber size and a disruption of tuber formation [56]. The role of 9-Lox in dormant potato tubers is unclear. Several studies investigated Lox activity in potato tubers during cold sweetening [54,57]. Lox was supposed to contribute to peroxidative damage occurring during cold storage. However, there was no clear correlation between cold sweetening and membrane permeability or lipid saturation level [54]. Furthermore, cold sweetening is a reversible process since sugar levels decrease when tubers are transferred back to higher temperatures, which is not in agreement with the irreversible peroxidation of membranes [3]. Although speculative at this point, we propose that patatin, PLA1 and Lox isoforms modulate in a cultivar specific manner the properties of membranes, which leads to genotypic differences in cold acclimation and sweetening by yet unknown mechanisms.

The adaptation of tubers to low temperature might include other pathways besides starch-sugar interconversion and membrane composition. Interesting candidates in this respect are the isoforms of a chloroplastic (plastidic) leucine aminopeptidase (LapA) which were more abundant in CIS-t than in CIS-s genotypes. The corresponding locus on chromosome XII consists of duplicated, physically closely linked *Lap* genes and is linked to QTL for tuber starch and sugar content. Plant leucine aminopeptidases are involved in a wide range of physiological processes [58]. LapA was first identified in potato in response to wounding and is assumed to be unique for Solanaceous plants [59]. LapA was induced by water deficit, salinity, pathogen attack and in response to jasmonic and abscisic acid [58,60,61]. The neutral *Lap* (*LapN*) was constitutively expressed in various plant species and is assumed to play a role in general cell maintenance, whereas expression of acidic *Lap* (*LapA*) was more specific [62]. Our findings suggest a novel regulatory function of LapA in starch-sugar interconversion. StLapA, presumably located in amyloplasts, may inhibit starch degradation by proteolysis of active enzymes, thereby indirectly repressing sugar accumulation.

Association analysis identified SNP_2746_ in the *StLapN* gene that was strongly associated with chip quality with and without cold storage, tuber starch content and starch yield. Attempts to score SNPs directly in the differentially expressed *StLapA* gene failed due to the presence of insertion-deletion polymorphisms in the amplicons derived from highly heterozygous tetraploid individuals. Since *StLapN* is separated from *StLapA* only by few kilobase pairs and since linkage disequilibrium (LD) in potato can extend over several hundred kilobase pairs [63], the association of SNP_2746_ with the tuber traits may be due to LD with *StLapA*. The average RSC of cultivars possessing the SNP allele *A*_*2746*_ was clearly lower than the average of cultivars lacking this allele throughout cold storage (Figure 4), which makes this SNP an attractive marker for breeding applications.

Differential spots were also assigned to proteins involved in metabolic processes like glycolysis or stress response. Heat shock proteins involved in stress responses were more abundant exclusively in the CIS-s genotypes. HSPs are ubiquitous molecular chaperones which protect other proteins from missfolding after temperature stresses. Accumulation of HSPs in response to cold stress has been reported [25,64,65]. Genotypic variation of HSPs can therefore have consequences for several metabolic processes in potato tubers including cold sweetening.

### Reducing sugar levels and tuber bruising

Intriguing overlaps were observed between the types of proteins that were differentially expressed in genotypes with high and low reducing sugar levels (this study) and genotypes with high and low susceptibility to tuber bruising [27]. Bruising describes the enzymatic discoloration of internal tuber tissue upon mechanical damage and seems biochemically unrelated with starch-sugar interconversion. In both studies however, protease inhibitors, patatins, lipoxygenase and phospholipase A1 (identical to lipase class III) were independently identified as genotype dependent differential proteins. In the case of Kunitz-type enzyme inhibitor S9C11, Lox and PLA1, the tryptic peptides matched unambiguously to the same loci (PGSC0003DMG400010147, PGSC0003DMG400020999 and PGSC0003DMG401031759), indicating that the differential proteins were products of the same genes, though the alleles associated with RSC and bruising susceptibility might not be the same. Susceptibility to bruising is strongly correlated with higher tuber starch content, and membrane stability may also play a role [66]. This suggests tuber starch content and/or membrane structure as common denominators of sugar levels and bruising, which influence both RSC and bruising susceptibility. PIs together with components of lipid metabolism and/or signaling might regulate both traits either directly or indirectly via regulation of tuber starch content or membrane composition.

## Conclusions

The combination of proteomics and association genetics described in this paper provides an innovative route to better understand complex plant traits at the molecular level, particularly in non model plants such as the potato. Comparative proteomics across a diversity panel enabled the identification of unapparent candidate genes not noticed in classical functional approaches, where protein abundance or activity in response to environmental changes is studied in a single genotype. Association of DNA polymorphisms in loci encoding differentially expressed proteins validated a role of leucine aminopeptidase in tuber starch content, starch yield and chip quality. Lap has not been considered so far as acting in carbohydrate metabolism or cold induced sweetening. The SNP’s associated with tuber quality can be used for marker-assisted selection of CIS resistant cultivars.

## Methods

### Plant material and tissue sampling

Field grown tubers of 39 cultivars and one breeding clone (Additional file 1: Table S1) were provided by Böhm-Nordkartoffel Agrarproduktion OHG (BNA, Ebstorf, Germany). Twenty cultivars had been selected for having good processing quality compared with twenty others having bad processing quality. After delivery, tubers were stored for twelve weeks at 4°C in the dark. Prior to and after one, two, four and twelve weeks of cold storage, tissue of three peeled tubers per cultivar and time point were individually snap frozen in liquid nitrogen. For protein extraction, frozen tuber tissue was ground to fine powder in a bead mill (Retsch, Haan, Germany) and stored at -80°C until use. Tissue aliquots were freeze-dried, for sugar extraction. For correlation analysis, total tuber proteins of all 40 cultivars without cold storage were separated by 2D-SDS-PAGE. For comparative proteome analysis during cold storage, tuber protein extracts from cultivars ‘Verdi’, ‘Lady Claire’, ‘Omega’, ‘Eurobeta’ and breeding clone 18 (pool “CIS-tolerant”, “CIS-t”), and ‘Elfe’, ‘Marabel’, ‘Solara’, ‘Melba’ and ‘Allians’ (pool “CIS-sensitive”, “CIS-s”) were pooled and analyzed. The population ‘CHIPS-ALL’ comprising 207 tetraploid genotypes was used for association mapping, which consisted of 33 standard varieties and 76, 91 and 7 breeding clones from Böhm-Nordkartoffel Agrarproduktion OHG (BNA, Ebstorf, Germany), Saka Pflanzenzucht GbR (Windeby, Germany) and Nordring-Kartoffelzucht-und Vermehrungs-GmbH (NORIKA, Groß Lüsewitz, Germany), respectively. This population has been evaluated in replicated field trials for chip color after harvest in autumn (without cold storage, CQA) and after three months storage at 4°C (CQS), for tuber yield (TY), starch content (TSC) and starch yield (TSY) [22].

### Glucose, fructose and sucrose measurement

Glucose, fructose and sucrose were estimated as described [17] with minor modifications. 100 mg dry powder were extracted with 1 ml ice cold 80% Ethanol and incubated at 80°C for 1 h. Extracts were concentrated to 100 μl using a vacuum concentrator (SpeedVac®, Thermo Fisher Scientific, MA, USA). 900 μl dH_2_O were added and samples were mixed until precipitates were dissolved. Saccharose, glucose and fructose were measured by a coupled enzymatic assay (“D-Glucose, D-Fructose, Saccharose”, R-Biopharm, Darmstadt, Germany) following the suppliers instructions. Sugar content was obtained as percent tuber dry weight (μg*100 μg^-1^ dry weight). Tuber reducing sugar content (RSC) resulted from the sum of glucose and fructose content. RSC was measured in three tubers per cultivar (biological replicates) and means and standard errors were estimated.

### Protein extraction

Total protein was extracted from 300 mg ground frozen tuber-tissue as described [29]. Protein pellets were re-suspended in urea-buffer (8 M urea, 2 M Thiourea). Protein abundance was estimated using the Qubit® protein assay kit (Invitrogen, CA, USA). The resulting protein extracts were frozen in liquid N_2_ and stored at -80°C until use.

### 2D-PAGE, image acquisition and data analysis

For correlation analysis between RSC and protein expression levels in 40 cultivars, protein of three individual tubers per cultivar was separated by analytical 2D-PAGE. 150 μg total tuber protein were subjected to isoelectric focusing (IEF) (Zoom® strips pH3-7NL, Invitrogen, CA, USA) using the Protean IEF system (BIORAD, CA, USA) according to the manufactures instructions. IPG (isoelectric focusing gel) strips were rehydrated for 12 h in 7 M urea, 2 M thiourea, 2% CHAPS (3-[(3-Cholamidopropyl) dimethylammonio]-1-propanesulfonate), 0.5% IPG-buffer pH 3–10 (Invitrogen, CA, USA), 25 mM DTT (Dithiothreitol) and trace amounts of bromphenol blue. Prior to SDS-PAGE IPG-strips were equilibrated at room temperature in 6 M urea, 0.375 M Tris–HCl pH 8.8, 2% SDS, 20% glycerol, 2% DTT for 10 min followed by a second equilibration in the same buffer except that DTT was replaced by 2.5% iodoacetamide. SDS-PAGE was run using 12% NuPAGE®Novex Bis-Tris gels (Invitrogen, CA, USA) in MES-SDS buffer system at 200 V. Gels were fixed for 1 h in 50% methanol, 10% acetic acid at room temperature followed by three washes with H_2_O. Gels were stained with PageBlue™ staining solution (Fermentas, St. Leon-Rot, Germany) according to the instructions of the manufacturer. Protein gels were digitalized using a daylight scanner integrated within the Proteineer spII spotting system (Bruker Daltonics, Bremen, Germany). Gels of three biological replicates per cultivar were analyzed. Protein spots were detected on scanned gels using the default spot detection settings in Proteome Weaver 2-DE analysis software package (BIORAD, CA, USA). Spot intensities were calculated subsequent to normalization of the spots using the pair-match-based normalization algorithm implemented in the software. Mean spot intensities and standard errors were calculated for 26 selected spots that were detectable in more than 70% of all gels of the cultivars and replicates. For correlation analysis, residuals of mean spot intensity and RSC were checked graphical for normal distribution and transformed into logarithmic values. Multiple R^2^ were calculated by regression analysis of logarithmic RSC values, respectively spot intensities and the genotype. Pearson’s product–moment correlation coefficient and significance were calculated using the SPSS software package (IBM, NY, USA). Spots showing significant correlations (p < 0.05) were isolated from 2D-gels and digested with trypsin. Tryptic peptides were identified by mass spectrometry (MS) and protein sequence database comparisons (see below). For comparative proteome analysis during cold storage, tuber protein extracts from cultivars ‘Verdi’, ‘Lady Claire’, ‘Omega’, ‘Eurobeta’ and breeding clone 18 (CIS-tolarant, CIS-t), and ‘Elfe’, ‘Marabel’, ‘Solara’, ‘Melba’ and ‘Allians’ (CIS-sensitive, CIS-s) were used. Total protein was extracted from three tubers per cultivar and time point prior to and after two, four and twelve weeks of storage at 4°C. Equimolar amounts of protein from a single tuber of CIS-t and CIS-s genotypes were combined, resulting in three pooled samples (biological replicates) per time point. 300 μg pooled protein was separated by 2D-PAGE using two different conditions for IEF and SDS-PAGE. IEF was performed using 24 cm IPG strips with immobilized pH gradients ranging from pH4-7 and pH 3–10 according to the manufacturer’s guidelines (GE Healthcare, WI, USA). IPG-strips were equilibrated (see above) and subjected to 16% SDS-PAGE (24 x 20 cm, 1 mm thickness) using the Tris-tricine buffer system [67] or 10% SDS-PAGE (24 × 20 cm, 1.5 mm thickness) using Tris-glycine buffer system [68]. Electrophoresis was carried out in the Ettan™DALTsix electrophoresis unit (GE Healthcare, WI, USA) at 30 V for 1 h followed by a constant current at 270 mA or using the PROTEAN Plus Dodeca Cell (BIORAD, CA, USA) at 30 V for 1 h, followed by a constant current at 200 mA. Electrophoresis was run for 16 h until the bromphenol-blue dye reached the bottom of the gel. Gels were fixed in 50% methanol/10% acetic acid for 2 h at room temperature and stained with PageBlue™ staining solution. Gel images were acquired as described above. Spots were detected using the default spot detection settings in the Delta2D analysis software (DECODON GmbH, Greifswald, Germany). Twenty four gel pictures (three biological replicates of pooled CIS-t and CIS-s genotypes at four time-points) were included in the analysis. Four analysis sets were composed of the gels generated before (T0) and after two, four and twelve weeks of cold storage. Delta2D analysis software was used to produce a fused image of the gels of one analysis set to identify qualitative and quantitative differences between CIS-t and CIS-s genotypes. Within one analysis set spot intensities were normalized by the vertical normalization algorithm implemented in the Delta2D software. The algorithm is based on the division of the spot intensity by the sum of all spot intensities on the respective gel. Significant differences in spot intensities between CIS-t and CIS-s were evaluated by statistical analysis implemented in the Delta2D software using the “between samples” T-Test assuming different group variances (Welch approximation) and without correction for multiple testing. Differentially expressed proteins were identified as described below.

### MS Analysis, MALDI Data Acquisition, Database Searching

Spots were selected from Coomassie-stained gels, excised and digested with trypsin using the Proteineer *spII* and *dp* systems (Bruker Daltonics, Bremen, Germany). Sample digests were spotted onto Anchorchip steel targets (Bruker Daltonics, Bremen, Germany) prepared with a thin layer of α-cyano-4-hydroxycinnamic acid (HCCA) matrix [69]. Peptide mass fingerprint (PMF) data were collected on an UltraflexIII MALDI ToF/ToF mass spectrometer (Bruker Daltonics, Bremen, Germany) and used for a first round of database searching using MASCOT 2.3 (http://www.matrixscience.com) against NCBI nr core collection and the potato genome sequencing consortium (PGSC) *Solanum tuberosum* group Phureja DM protein database. PMF searches were initially performed with a mass tolerance set to 50 ppm and re-performed with a tolerance of 150 ppm when post-calibration based on tryptic self-digest peptides failed.

Following sample recrystallization, LIFT MS/MS spectra were collected on up to five precursors selected from the PMF on the basis of peak intensity and relative isolation [70]. MS/MS data were used to search both databases with a mass tolerance of 0.4 Da for both parent and fragment masses of MS/MS searches. For both MS and MS/MS, MASCOT scores falling below the 95% certainty criterion for the respective database were not considered significant.

Genomic positions of genes corresponding to the identified proteins were identified based on blastn and tblastn searches of the potato [30] and tomato [71] genome sequences (http://solanaceae.plantbiology.msu.edu/integrated_searches.shtml, http://solgenomics.net/tools/blast/index.pl).

### Generation of SNP markers

Locus specific amplicons were generated by PCR (polymerase chain reaction) using genomic DNA of the individuals of the CHIPS-ALL population as template. A 1150 bp DNA fragment comprising the ‘putative Kunitz-type tuber invertase inhibitor’ (KT-InvInh, PGSC0003DMG400010146) was amplified with the forward (5´-TCAATAGAATTCCTTACCCG-3´) and reverse (5´-CAATAACGATATCTAGACCTGATTAAC-3´) primers at 55°C annealing temperature. An 850 bp DNA fragment from ‘neutral leucine aminopeptidase’ (*LapN*, superscaffold PGSC0003DMB000000116-chr12:910581..2552409) was amplified using the forward (5´-GCTTCCTGGTCTTGGCTCA-3´) and reverse (5´-GCAGCCAGGTCAGAAATCAA-3´) primers at 60°C annealing temperature. Standard PCR conditions were: 25 μL reaction mixture containing 50 ng genomic DNA in 1× Ammonium PCR buffer (Ampliqon, Scovlunde, Denmark), 2.5 mM MgCl_2_, 0.2 mM of each dNTP, 5 μM each forward and reverse primer and 1 U Taq polymerase (Ampliqon, Scovlunde, Denmark); 4 min initial denaturation at 94°C; 34 cycles of 45 sec denaturation at 94°C, 30 sec annealing at 55°C, 75 sec elongation at 72°C; final elongation at 72°C for 6 min. Amplicons were custom sequenced at the Max-Planck Genome Center Cologne using the dideoxy chain-termination sequencing method, an ABI PRISM Dye Terminator Cycle Sequencing Ready Reaction Kit and an ABI PRISM 3730 automated DNA Sequencer (Applied Biosystems, Weiterstadt, Germany). KT-InvInh amplicons were sequenced with the internal reverse primer 5´-CTACGCCTTGATGAACACAAATG-3′ and LapN amplicons were sequenced with the forward primer. Single nucleotide polymorphisms (SNP) in amplicon sequences were detected and scored as described [72].

### Association analysis

The CHIPS-ALL population used for association analysis has been described [22]. It comprised 209 tetraploid cultivars from three commercial potato breeding programs and 34 standard varieties, which have been phenotyped in replicated field trials for tuber starch content (TSC), tuber yield (TY), starch yield (TSY), chip quality in autumn before cold storage (CQA) and chip quality after 3–4 months storage at 4°C (CQS). No significant population substructure has been detectable in the CHIPS-ALL population. Major marker-trait associations (p < 0.001) have been consistent across different models for association analysis [22]. The new SNP markers were tested for association with TSC, TY, TSY, CQA and CQS based on the same model as used previously [22] using the GLM procedure in SPSS (SPSS GmbH Software, München, Germany):

y*=origin+marker+error

y* represent the adjusted trait means. ‘Origin’ is a fixed factor with four classes, which corresponds to the origin of each cultivar from one of three breeding programs or standards. ‘Marker’ is a fixed factor with five levels corresponding to the five marker genotypic classes of a bi-allelic SNP (*A*_*1*_*A*_*1*_*A*_*1*_*A*_*1*_*, A*_*1*_*A*_*1*_*A*_*1*_*A*_*2*_*, A*_*1*_*A*_*1*_*A*_*2*_*A*_*2*_*, A*_*1*_*A*_*2*_*A*_*2*_*A*_*2*_*and A*_*2*_*A*_*2*_*A*_*2*_*A*_*2*_).

Analysis of variance (ANOVA) was used to detect significant differences between RSC means of the genotype groups with the SNP allele *StLapN-SNP*_*2746*_ present or absent in the 40 cultivars.

## Abbreviations

CIS: Cold induced sweetening; RSC: Reducing sugar content; QTL: Quantitative trait loci; CQA: Chip quality after harvest; CQS: Chip quality after cold storage; PCR: Polymerase chain reaction; TY: Tuber yield; TSC: Tuber starch content; SNP: Single nucleotide polymorphism.

## Competing interests

The authors have declared no conflict of interests.

## Authors’ contributions

MF carried out protein extraction, 2D-PAGE, image aquisition, data analysis and drafted the manuscript. LS analyzed genomic organization of Lap and KT-InvInh, generated SNP markers and performed association analysis with tuber quality traits. TC and JS performed MS analysis, MALDI data acquisition and database searching. MK participated in protein extraction, estimation of tuber sugar concentration, 2D-PAGE and image acquisition. ET and HRH provided plant material and participated in design of this study. CG conceived the study, participated in its design and coordination, carried out statistical analyses and drafted the manuscript. All authors read and approved the final manuscript.

## Supplementary Material

Additional file 1: Table S1 Cultivars used for comparative proteome profiling. Click here for file

Additional file 2: Table S2 Data on mean RSC and spot intensities from 40 cultivars and 26 selected protein spots for correlation analysis. Click here for file

Additional file 3: Table S3 Data on protein identification from 5 protein spots correlated with RSC. Click here for file

Additional file 4: Table S4 Differentially expressed tuber proteins in genotype pool CIS-t versus pool CIS-s during 12 weeks of storage at 4°C. Click here for file

Additional file 5: Table S5 Data on protein identification from differentially expressed tuber proteins in genotype pool CIS-t versus pool CIS-s during 12 weeks of storage at 4°C. Click here for file

Additional file 6: Figure S1 Positions of SNPs and indels in the amplicons used for association analysis. Position refers to the start codon (A_1_TG). Primer sequences are underlined. Exons are shaded grey. Click here for file

## References

[B1] Müller-Thurgau H Über Zuckeranhäufung in Pflanzenteilen in Folge niederer Temperatur Landwirtschaftliches Jahrbuch 1882 11 751 828

[B2] Guy CL Huber JLA Huber SC Sucrose phosphate synthase and sucrose accumulation at low-temperature Plant Physiol 1992 100 1 502 508 10.1104/pp.100.1.502 16652990 PMC1075578

[B3] Sowokinos JR Biochemical and molecular control of cold-induced sweetening in potatoes Am J Potato Res 2001 78 3 221 236 10.1007/BF02883548

[B4] Malone JG Mittova V Ratcliffe RG Kruger NJ The response of carbohydrate metabolism in potato tubers to low temperature Plant Cell Physiol 2006 47 9 1309 1322 10.1093/pcp/pcj101 16936336

[B5] Geigenberger P Regulation of sucrose to starch conversion in growing potato tubers J Exp Bot 2003 54 382 457 465 10.1093/jxb/erg074 12508056

[B6] Kotting O Kossmann J Zeeman SC Lloyd JR Regulation of starch metabolism: the age of enlightenment? Curr Opin Plant Biol 2010 13 3 321 329 20171927 10.1016/j.pbi.2010.01.003

[B7] Geigenberger P Regulation of starch biosynthesis in response to a fluctuating environment Plant Physiol 2011 155 4 1566 1577 10.1104/pp.110.170399 21378102 PMC3091114

[B8] Nielsen TH Deiting U Stitt M A [beta]-amylase in potato tubers is induced by storage at low temperature Plant Physiol 1997 113 2 503 510 12223621 10.1104/pp.113.2.503 PMC158166

[B9] Zrenner R Schuler K Sonnewald U Soluble acid invertase determines the hexose-to-sucrose ratio in cold-stored potato tubers Planta 1996 198 2 246 252 8580777 10.1007/BF00206250

[B10] Sowokinos JR Vigdorovich V Abrahamsen M Molecular cloning and sequence variation of UDP-glucose pyrophosphorylase cDNAs from potatoes sensitive and resistant to cold sweetening J Plant Physiol 2004 161 8 947 955 10.1016/j.jplph.2004.04.006 15384406

[B11] Brummell DA Chen RK Harris JC Zhang H Hamiaux C Kralicek AV McKenzie MJ Induction of vacuolar invertase inhibitor mRNA in potato tubers contributes to cold-induced sweetening resistance and includes spliced hybrid mRNA variants J Exp Bot 2011 62 10 3519 3534 10.1093/jxb/err043 21393382 PMC3130176

[B12] Greiner S Rausch T Sonnewald U Herbers K Ectopic expression of a tobacco invertase inhibitor homolog prevents cold-induced sweetening of potato tubers Nat Biotechnol 1999 17 7 708 711 10.1038/10924 10404166

[B13] McKenzie MJ Chen RK Harris JC Ashworth MJ Brummell DA Post-translational regulation of acid invertase activity by vacuolar invertase inhibitor affects resistance to cold-induced sweetening of potato tubers Plant Cell Environ 2013 36 1 176 185 10.1111/j.1365-3040.2012.02565.x 22734927

[B14] Pressey R Invertase inhibitor from potatoes: purification, characterization, and reactivity with plant invertases Plant Physiol 1967 42 12 1780 1786 10.1104/pp.42.12.1780 16656719 PMC1086796

[B15] Glaczinski H Heibges A Salamini R Gebhardt C Members of the Kunitz-type protease inhibitor gene family of potato inhibit soluble tuber invertase in vitro Potato Res 2002 45 2–3 163 176

[B16] Dale MF Bradshaw JE Progress in improving processing attributes in potato Trends Plant Sci 2003 8 7 310 312 10.1016/S1360-1385(03)00130-4 12878011

[B17] Menendez CM Ritter E Schäfer-Pregl R Walkemeier B Kalde A Salamini F Gebhardt C Cold sweetening in diploid potato: mapping quantitative trait loci and candidate genes Genetics 2002 162 3 1423 1434 12454085 10.1093/genetics/162.3.1423 PMC1462350

[B18] Coffin RH Yada RY Parkin KL Grodzinski B Stanley DW Effect of Low-Temperature Storage on Sugar Concentrations and Chip Color of Certain Processing Potato Cultivars and Selections J Food Sci 1987 52 3 639 645 10.1111/j.1365-2621.1987.tb06692.x

[B19] Gebhardt C Menendez CM Chen X Li L Schäfer-Pregl R Salamini F Genomic approaches for the improvement of tuber quality traits in potato Acta Hortic 2005 684 85 92

[B20] Chen X Salamini F Gebhardt C A potato molecular-function map for carbohydrate metabolism and transport Theor Appl Genet 2001 102 2 284 295

[B21] Schäfer-Pregl R Ritter E Concilio L Hesselbach J Lovatti L Walkemeier B Thelen H Salamini F Gebhardt C Analysis of quantitative trait loci (QTLs) and quantitative trait alleles (QTAs) for potato tuber yield and starch content Theor Appl Genet 1998 97 5 834 846

[B22] Li L Paulo MJ Strahwald J Lübeck J Hofferbert HR Tacke E Junghans H Wunder J Draffehn A Van Eeuwijk F Natural DNA variation at candidate loci is associated with potato chip color, tuber starch content, yield and starch yield Theor Appl Genet 2008 116 8 1167 1181 10.1007/s00122-008-0746-y 18379755 PMC2358939

[B23] Li L Strahwald J Hofferbert HR Lubeck J Tacke E Junghans H Wunder J Gebhardt C DNA variation at the invertase locus invGE/GF is associated with tuber quality traits in populations of potato breeding clones Genetics 2005 170 2 813 821 10.1534/genetics.104.040006 15802505 PMC1450405

[B24] Draffehn AM Meller S Li L Gebhardt C Natural diversity of potato (*Solanum tuberosum*) invertases BMC Plant Biol 2010 10 271 10.1186/1471-2229-10-271 21143910 PMC3012049

[B25] Bagnaresi P Moschella A Beretta O Vitulli F Ranalli P Perata P Heterologous microarray experiments allow the identification of the early events associated with potato tuber cold sweetening BMC Genomics 2008 9 176 10.1186/1471-2164-9-176 18416834 PMC2358903

[B26] De Vienne D Leonardi A Damerval C Zivy M Genetics of proteome variation for QTL characterization: application to drought-stress responses in maize J Exp Bot 1999 50 332 303 309

[B27] Urbany C Colby T Stich B Schmidt L Schmidt J Gebhardt C Analysis of natural variation of the potato tuber proteome reveals novel candidate genes for tuber bruising J Proteome Res 2012 11 2 703 716 10.1021/pr2006186 22047174

[B28] Yang Y Qiang X Owsiany K Zhang S Thannhauser TW Li L Evaluation of different multidimensional LC-MS/MS pipelines for isobaric tags for relative and absolute quantitation (iTRAQ)-based proteomic analysis of potato tubers in response to cold storage J Proteome Res 2011 10 10 4647 4660 10.1021/pr200455s 21842911

[B29] Hoehenwarter W Van Dongen JT Wienkoop S Steinfath M Hummel J Erban A Sulpice R Regierer B Kopka J Geigenberger P A rapid approach for phenotype-screening and database independent detection of cSNP/protein polymorphism using mass accuracy precursor alignment Proteomics 2008 8 20 4214 4225 10.1002/pmic.200701047 18924179

[B30] Potato Genome Sequencing Consortium Genome sequence and analysis of the tuber crop potato Nature 2011 475 7355 189 195 10.1038/nature10158 21743474

[B31] Odeny DA Stich B Gebhardt C Physical organization of mixed protease inhibitor gene clusters, coordinated expression and association with resistance to late blight at the *StKI* locus on potato chromosome III Plant, Cell & Env 2010 33 12 2149 2161 10.1111/j.1365-3040.2010.02213.x 20716067

[B32] Gebhardt C Walkemeier B Henselewski H Barakat A Delseny M Stuber K Comparative mapping between potato (Solanum tuberosum) and Arabidopsis thaliana reveals structurally conserved domains and ancient duplications in the potato genome Plant Journal 2003 34 4 529 541 10.1046/j.1365-313X.2003.01747.x 12753591

[B33] Pressey R Shaw R Effect of temperature on invertase, invertase inhibitor, and sugars in potato tubers Plant Physiol 1966 41 10 1657 1661 10.1104/pp.41.10.1657 16656454 PMC550589

[B34] Richardson DL Davies HV Ross HA Mackay GR Invertase activity and its relation to hexose accumulation in potato-tubers J Exp Bot 1990 41 222 95 99

[B35] Pressey R Role of invertase in accumulation of sugars in cold-stored potatoes Am Potato J 1969 46 8 291

[B36] Bhaskar PB Wu L Busse JS Whitty BR Hamernik AJ Jansky SH Buell CR Bethke PC Jiang J Suppression of the vacuolar invertase gene prevents cold-induced sweetening in potato Plant Physiol 2010 154 2 939 948 10.1104/pp.110.162545 20736383 PMC2948980

[B37] Schwimmer S Makower RU Rorem ES Invertase & invertase inhibitor in potato Plant Physiol 1961 36 3 313 316 10.1104/pp.36.3.313 16655511 PMC406138

[B38] Greiner S Krausgrill S Rausch T Cloning of a tobacco apoplasmic invertase inhibitor Plant Physiol 1998 116 2 733 742 10.1104/pp.116.2.733 9489020 PMC35133

[B39] Liu X Song B Zhang H Li XQ Xie C Liu J Cloning and molecular characterization of putative invertase inhibitor genes and their possible contributions to cold-induced sweetening of potato tubers Mol Genet Genomics 2010 284 3 147 159 10.1007/s00438-010-0554-3 20617340

[B40] Lehesranta SJ Davies HV Shepherd LV Koistinen KM Massat N Nunan N McNicol JW Karenlampi SO Proteomic analysis of the potato tuber life cycle Proteomics 2006 6 22 6042 6052 10.1002/pmic.200600383 17106910

[B41] Bauw G Nielsen HV Emmersen J Nielsen KL Jorgensen M Welinder KG Patatins, Kunitz protease inhibitors and other major proteins in tuber of potato cv. Kuras Febs J 2006 273 15 3569 3584 10.1111/j.1742-4658.2006.05364.x 16884497

[B42] Jorgensen M Bauw G Welinder KG Molecular properties and activities of tuber proteins from starch potato cv. Kuras J Agric Food Chem 2006 54 25 9389 9397 10.1021/jf0623945 17147423

[B43] Heibges A Glaczinski H Ballvora A Salamini F Gebhardt C Structural diversity and organization of three gene families for Kunitz-type enzyme inhibitors from potato tubers (Solanum tuberosum L.) Mol Genet Genomics 2003 269 4 526 534 10.1007/s00438-003-0860-0 12783302

[B44] Pouvreau L Gruppen H Piersma SR van den Broek LA Van Koningsveld GA Voragen AG Relative abundance and inhibitory distribution of protease inhibitors in potato juice from cv. Elkana J Agric Food Chem 2001 49 6 2864 2874 10.1021/jf010126v 11409980

[B45] Ryan CA Protease inhibitors in plants - genes for improving defenses against insects and pathogens Annu Rev Phytopathol 1990 28 425 449 10.1146/annurev.py.28.090190.002233

[B46] Heibges A Salamini F Gebhardt C Functional comparison of homologous members of three groups of Kunitz-type enzyme inhibitors from potato tubers (Solanum tuberosum L.) Mol Genet Genomics 2003 269 4 535 541 10.1007/s00438-003-0861-z 12783303

[B47] Ishikawa A Ohta S Matsuoka K Hattori T Nakamura K A family of potato genes that encode Kunitz-type proteinase inhibitors: structural comparisons and differential expression Plant Cell Physiol 1994 35 2 303 312 8069493

[B48] Svendsen I Hejgaard J Mundy J Complete amino-acid-sequence of the alpha-amylase/subtilisin inhibitor from barley Carlsberg Res Comm 1986 51 1 43 50 10.1007/BF02907994

[B49] Andrews DL Beames B Summers MD Park WD Characterization of the lipid acyl hydrolase activity of the major potato (*Solanum tuberosum*) tuber protein, patatin, by cloning and abundant expression in a baculovirus vector Biochem J 1988 252 1 199 206 3048246 10.1042/bj2520199 PMC1149124

[B50] Hirschberg HJ Simons JW Dekker N Egmond MR Cloning, expression, purification and characterization of patatin, a novel phospholipase A Eur J Biochem 2001 268 19 5037 5044 10.1046/j.0014-2956.2001.02411.x 11589694

[B51] Scherer GFE Ryu SB Wang XM Matos AR Heitz T Patatin-related phospholipase A: nomenclature, subfamilies and functions in plants Trends Plant Sci 2010 15 12 693 700 10.1016/j.tplants.2010.09.005 20961799

[B52] Theocharis A Clement C Barka EA Physiological and molecular changes in plants grown at low temperatures Planta 2012 235 6 1091 1105 10.1007/s00425-012-1641-y 22526498

[B53] Ruelland E Vaultier MN Zachowski A Hurry V Cold Signalling and Cold Acclimation in Plants Adv Bot Res 2009 49 35 150

[B54] Fauconnier ML Rojas-Beltran J Delcarte J Dejaeghere F Marlier M Du Jardin P Lipoxygenase pathway and membrane permeability and composition during storage of potato tubers (Solanum tuberosum L. cv Bintje and Desiree) in different conditions Plant Biology 2002 4 1 77 85 10.1055/s-2002-20439

[B55] Royo J Vancanneyt G Perez AG Sanz C Stormann K Rosahl S SanchezSerrano JJ Characterization of three potato lipoxygenases with distinct enzymatic activities and different organ-specific and wound-regulated expression patterns J Biol Chem 1996 271 35 21012 21019 10.1074/jbc.271.35.21012 8702864

[B56] Kolomiets MV Hannapel DJ Chen H Tymeson M Gladon RJ Lipoxygenase is involved in the control of potato tuber development Plant Cell 2001 13 3 613 626 11251100 10.1105/tpc.13.3.613 PMC135504

[B57] Wismer WV Worthing WM Yada RY Marangoni AG Membrane lipid dynamics and lipid peroxidation in the early stages of low-temperature sweetening in tubers of Solanum tuberosum Physiol Plant 1998 102 3 396 410 10.1034/j.1399-3054.1998.1020308.x

[B58] Chao WS Gu YQ Pautot V Bray EA Walling LL Leucine aminopeptidase RNAs, proteins, and activities increase in response to water deficit, salinity, and the wound signals systemin, methyl jasmonate, and abscisic acid Plant Physiol 1999 120 4 979 992 10.1104/pp.120.4.979 10444081 PMC59357

[B59] Hildmann T Ebneth M Penacortes H Sanchezserrano JJ Willmitzer L Prat S General Roles of Abscisic and Jasmonic Acids in Gene Activation as a Result of Mechanical Wounding Plant Cell 1992 4 9 1157 1170 1392612 10.1105/tpc.4.9.1157 PMC160206

[B60] Pautot V Holzer FM Reisch B Walling LL Leucine aminopeptidase: an inducible component of the defense response in *Lycopersicon esculentum* (tomato) Proc Natl Acad Sci USA 1993 90 21 9906 9910 10.1073/pnas.90.21.9906 8234334 PMC47681

[B61] Gu YQ Pautot V Holzer FM Walling LL A complex array of proteins related to the multimeric leucine aminopeptidase of tomato Plant Physiol 1996 110 4 1257 1266 12226257 10.1104/pp.110.4.1257 PMC160919

[B62] Chao WS Pautot V Holzer FM Walling LL Leucine aminopeptidases: the ubiquity of LAP-N and the specificity of LAP-A Planta 2000 210 4 563 573 10.1007/s004250050045 10787049

[B63] Achenbach U Paulo J Ilarionova E Lubeck J Strahwald J Tacke E Hofferbert HR Gebhardt C Using SNP markers to dissect linkage disequilibrium at a major quantitative trait locus for resistance to the potato cyst nematode *Globodera pallida* on potato chromosome V Theor Appl Genet 2009 118 3 619 629 10.1007/s00122-008-0925-x 19020852

[B64] Bae MS Cho EJ Choi EY Park OK Analysis of the Arabidopsis nuclear proteome and its response to cold stress Plant J 2003 36 5 652 663 10.1046/j.1365-313X.2003.01907.x 14617066

[B65] Kawamura Y Uemura M Mass spectrometric approach for identifying putative plasma membrane proteins of Arabidopsis leaves associated with cold acclimation Plant J 2003 36 2 141 154 10.1046/j.1365-313X.2003.01864.x 14535880

[B66] Urbany C Stich B Schmidt L Simon L Berding H Junghans H Niehoff KH Braun A Tacke E Hofferbert HR Association genetics in *Solanum tuberosum* provides new insights into potato tuber bruising and enzymatic tissue discoloration BMC Genomics 2011 12 7 10.1186/1471-2164-12-7 21208436 PMC3023753

[B67] Schagger H Tricine-SDS-PAGE Nat Protoc 2006 1 1 16 22 10.1038/nprot.2006.4 17406207

[B68] Laemmli UK Cleavage of Structural Proteins during Assembly of Head of Bacteriophage-T4 Nature 1970 227 5259 5680 10.1038/227680a0 5432063

[B69] Gobom J Schuerenberg M Mueller M Theiss D Lehrach H Nordhoff E alpha-cyano-4-hydroxycinnamic acid affinity sample preparation. A protocol for MALDI-MS peptide analysis in proteomics Anal Chem 2001 73 3 434 438 10.1021/ac001241s 11217742

[B70] Suckau D Resemann A Schuerenberg M Hufnagel P Franzen J Holle A A novel MALDI LIFT-TOF/TOF mass spectrometer for proteomics Anal Bioanal Chem 2003 376 7 952 965 10.1007/s00216-003-2057-0 12830354

[B71] Tomato Genome Sequencing Consortium The tomato genome sequence provides insights into fleshy fruit evolution Nature 2012 485 7400 635 641 10.1038/nature11119 22660326 PMC3378239

[B72] Pajerowska-Mukhtar K Stich B Achenbach U Ballvora A Lubeck J Strahwald J Tacke E Hofferbert HR Ilarionova E Bellin D Single nucleotide polymorphisms in the allene oxide synthase 2 gene are associated with field resistance to late blight in populations of tetraploid potato cultivars Genetics 2009 181 3 1115 1127 10.1534/genetics.108.094268 19139145 PMC2651047

