# Supplementary material for: Novel candidate genes influencing natural variation in potato tuber cold sweetening identified by comparative proteomics and association mapping
Source: BMC Plant Biol. 2013 Aug 7;13:113. doi: 10.1186/1471-2229-13-113 (PMC3750364; doi:10.1186/1471-2229-13-113)
Supplement: Additional file 1: Table S1 — Cultivars used for comparative proteome profiling. [file 1471-2229-13-113-S1.docx]

|  | **Code** | **Cultivar** | **Identification no. ^c^** | **Breeder ^c^** | **Registration ^c^** |
| --- | --- | --- | --- | --- | --- |
| High chip quality | 1 | Agria | 2539 | Kartoffelzucht Böhm | 1985 |
|  | 2 | Afra | 2741 | Dr. H Böhm, G. Henhold | 1990 |
|  | 3 | Topas | 3552 | Böhm-Nordkartoffel Agrarproduktion | 2005 |
|  | 4 | Lady Claire ^a^ | 3562 | C. Meijer B.V. | 2009 |
|  | 5 | Tomensa | 2731 | Nordkartoffel Zuchtgesellschauft | 1989 |
|  | 6 | Goldika | 3208 | Böhm-Nordkartoffel Agrarproduktion | 2001 |
|  | 7 | Omega ^a^ | 3498 | Böhm-Nordkartoffel Agrarproduktion | 2004 |
|  | 8 | Roberta | 3408 | Saatzucht Rudolf Pohl | 2002 |
|  | 9 | Innovator | 3561 | HZPC Holland B.V. | 2009 |
|  | 10 | Eurobeta ^a^ | 3703 | Böhm-Nordkartoffel Agrarproduktion | 2008 |
|  | 11 | Rumba | 3776 | Böhm-Nordkartoffel Agrarproduktion | 2010 |
|  | 12 | Antina | 3549 | Böhm-Nordkartoffel Agrarproduktion | 2009 |
|  | 13 | Europrima | 3589 | Böhm-Nordkartoffel Agrarproduktion | 2006 |
|  | 14 | Osira | 3750 | Böhm-Nordkartoffel Agrarproduktion | 2009 |
|  | 15 | Pirol | 3334 | NORIKA | 2000 |
|  | 16 | Fontane | 3621 | Lantmännen SW Seed B.V. | 2008 |
|  | 17 | Verdi ^a^ | 3454 | Uniplanta-Saatzucht | 2003 |
|  | 18 | Breeding clone 18 ^a^ | - | Böhm-Nordkartoffel Agrarproduktion | - |
|  | 19 | Opal | 3489 | Pflanzenzucht Saka | 2004 |
|  | 20 | Golf | 3453 | Uniplanta-Saatzucht | 2003 |
| Low chip quality | 21 | Cilena | 2364 | Nordkartoffel Zuchtgesellschauft | 1981 |
|  | 22 | Elfe ^b^ | 3456 | Nordkartoffel Zuchtgesellschauft | 2003 |
|  | 23 | Arnika | 2668 | Pflanzenzucht Saka | 1988 |
|  | 24 | Marabel^b^ | 2898 | Kartoffelzucht Böhm | 1993 |
|  | 25 | Milva | 3230 | Hergen Berding | 2009 |
|  | 26 | Allians ^b^ | 3566 | Böhm-Nordkartoffel Agrarproduktion | 2009 |
|  | 27 | Belana | 3340 | H. Berding | 2000 |
|  | 28 | Red Fantasy | 3588 | Böhm-Nordkartoffel Agrarproduktion | 2006 |
|  | 29 | Solara ^b^ | 2728 | Nordkartoffel Zuchtgesellschauft | 1989 |
|  | 30 | Satina | 2885 | Pflanzenzucht Saka | 1993 |
|  | 31 | Albatros | 3160 | NORIKA | 1996 |
|  | 32 | Sibu | 2883 | Pflanzenzucht Saka | 1993 |
|  | 33 | Amado | 3420 | Böhm-Nordkartoffel Agrarproduktion | 2002 |
|  | 34 | Solist | 3312 | NORIKA | 1999 |
|  | 35 | Christa | 2105 | KWS SAAT AG | 1975 |
|  | 36 | Tomba | 3059 | Nordkartoffel Zuchtgesellschauft | 1995 |
|  | 37 | Kuras | 3236 | Kartoffelzucht Böhm | 2002 |
|  | 38 | Melba ^b^ | 3773 | Böhm-Nordkartoffel Agrarproduktion | 2009 |
|  | 39 | Filea | 2879 | Nordkartoffel Zuchtgesellschauft | 1993 |
|  | 40 | Concordia | 3702 | Böhm-Nordkartoffel Agrarproduktion | 2008 |
| **^a^** Cultivars included in the CIS-tolerant (CIS-t) pool  **^b^** Cultivars included in the CIS-sensitive (CIS-s) pool  **^c^** Information according to the ‘Beschreibende Sortenliste Kartoffeln’ (Bundessortenamt, Hannover, Germany, 2010/2011) | | | | | |

**Supplementary Table S1 - Cultivars used for comparative proteome profiling.**
