# Supplementary material for: Novel candidate genes influencing natural variation in potato tuber cold sweetening identified by comparative proteomics and association mapping
Source: BMC Plant Biol. 2013 Aug 7;13:113. doi: 10.1186/1471-2229-13-113 (PMC3750364; doi:10.1186/1471-2229-13-113)
Supplement: Additional file 4: Table S4 — Differentially expressed tuber proteins in genotype pool CIS-t versus pool CIS-s during 12 weeks of storage at 4°C. [file 1471-2229-13-113-S4.docx]

**Supplementary Table S4 - Differentially expressed proteins in genotype pools CIS-t and CIS-s during 12 weeks of storage at 4°C**

| **Spot No** | **Protein** | **Accession no.** | **Locus**  **PGSC0003DMG ^1^** | **Superscaffold**  **PGSC0003DMB** | **Chromosome** | **Mean spot volume ±SE ^1^** |
| --- | --- | --- | --- | --- | --- | --- |
|  | **Protease Inhibitors** |  |  |  |  |  |
| 2 | Serine protease inhibitor 7 | P30941.2 | 400010128 | 000000159 | III |  |
| 4 | Serine protease inhibitor 7 | P30941.2 | 400010128 | 000000159 | III |  |
| 3 | Kunitz-type enzyme inhibitor S9C11 | AAL67830.1 | 400010147 | 000000159 | III |  |
| 6 | putative miraculin | CAC40756.1 | 400008546 | 000000314 | XII |  |
| 14 | Aspartic protease inhibitor 5 | P58519.1 | 400009511  400009513 | 000000400 | III |  |
| 10 | Kunitz-type proteinase inhibitor | AAM21645.1 | 400010128, 400009512 | 000000159 000000400 | III |  |
| 11 | Kunitz-type proteinase inhibitor | AAM21645.1 | 400010128, 400009512 | 000000159 000000400 | III |  |
| 12 | Kunitz-type proteinase inhibitor | AAM21645.1 | 400010128, 400009512 | 000000159 000000400 | III |  |
| 19 | putative miraculin | CAC40756.1 | 400010170 | 000000400 | III |  |
| 20 | Cysteine protease inhibitor 1 | P20347.3 | 400010134 | 000000159 | III |  |
| 21 | Proteinase inhibitor II | CAA27730 | 400004547 400004548 | 000000159 | III |  |
| 22 | Proteinase inhibitor II | CAA27730 | 400004547 400004548 | 000000400 | III |  |
| 24 | Proteinase inhibitor II | CAA27730.1 | 400004547 | 000000400 | III |  |
|  | **Storage proteins** |  |  |  |  |  |
| 1 | Patatin-2-Kuras 4 | Q3YJT0 | 400014104 | 000000402  000000779 | VIII |  |
| 13 | Patatin Group M-3 | Q2MY51 | 400008749 | 000000402 | VIII |  |
| 44 | Patatin-3-Kuras 1 | Q3YJS9.1 | 401017090 | 000000779 | VIII |  |
| 45 | Patatin-3-Kuras 1 | Q3YJS9.1 | 401017090 | 000000779 | VIII |  |
| 46 | Patatin-3-Kuras 1 | Q3YJS9.1 | 401017090 | 000000779 | VIII |  |
|  | **Lipid metabolism** |  |  |  |  |  |
| 31 | Lipoxygenase | X95512.1 | 400020999 | 000000147  000000208 | VIII |  |
| 32 | Lipoxygenase | X95512.1 | 400020999 | 000000147  000000208 | VIII |  |
| 33 | Lipoxygenase | X95512.1 | 400020999 | 000000147  000000208 | VIII |  |
| 34 | Lipoxygenase | X95512.1 | 400020999 | 000000147  000000208 | VIII |  |
| 35 | Lipoxygenase | X95512.1 | 400020999 | 000000147  000000208 | VIII |  |
| 36 | Phospholipase A1 | ABQ95989.1 | 401031759 | 000000476 | II |  |
| 39 | Phospholipase A1 | ABQ95989.1 | 401031759 | 000000476 | II |  |
| 40 | Phospholipase A1 | ABQ95989.1 | 401031759 | 000000476 | II |  |
|  | **Glycolysis** |  |  |  |  |  |
| 43 | Phosphoglycerate kinase | ABB87110 | 400022119 | 000000076 | VII |  |
|  | **Heat shock proteins** |  |  |  |  |  |
| 5 | chloroplast small heat shock protein class I | AAQ19680.1 | 400011631  400002928  400011632  400011628  400011630 | 000000332 | IX |  |
| 27 | Heat shock protein (HSP70) | XP_002512741 | 400014212 400027750  400019208 400000398 400000444 400030405 400008917 | 000000026  000000577  000000106  000000101  000000101  000000128  000000021 | III  ?  X  XI  XI  ?  IX |  |
| 28 | 101 kDa heat shock protein | AAC83688.2 | 400024644 | 000000062 | III |  |
|  | **Proteases** |  |  |  |  |  |
| 47 | Leucine aminopeptidase, chloroplastic | P31427.2 | - | 000000116 | XII |  |
| 48 | Leucine aminopeptidase, chloroplastic | P31427.2 | - | 000000116 | XII |  |
| 49 | Leucine aminopeptidase, chloroplastic | P31427.2 | - | 000000116 | XII |  |
| 50 | Leucine aminopeptidase, chloroplastic | P31427.2 | - | 000000116 | XII |  |
|  | **Pathogenesis related proteins** |  |  |  |  |  |
| 17 | putative PR-10 type pathogenesis-related protein | BAJ25784.1 | 400033354 400002414 400023236 400030255 | 000001304  000000949  000000744  000000708 | IV  IV  ?  III |  |
|  | **Cytoskeleton** |  |  |  |  |  |
| 30 | Actin | BAK57343.1 | 400000439  400018449  400003985  400023429  400027746  400023708 | 000000101  000000192  000000131  000000072  000000577  000000129 | ?  III  XI  V  ?  X |  |
|  | **Unidentified proteins** |  |  |  |  |  |
| 7 | not identified |  |  |  |  |  |
| 8 | not identified |  |  |  |  |  |
| 9 | not identified |  |  |  |  |  |
| 15 | not identified |  |  |  |  |  |
| 16 | not identified |  |  |  |  |  |
| 18 | not identified |  |  |  |  |  |
| 23 | not identified |  |  |  |  |  |
| 25 | not identified |  |  |  |  |  |
| 26 | not identified |  |  |  |  |  |
| 29 | not identified |  |  |  |  |  |
| 37 | not identified |  |  |  |  |  |
| 38 | not identified |  |  |  |  |  |
| 41 | not identified |  |  |  |  |  |
| 42 | not identified |  |  |  |  |  |

**^1^** Bar-charts show the means and standard errors (SE) of protein spot volumes obtained from three replicates of 2D protein patterns of pooled CIS-t (grey bars) and CIS-s (black bars) genotypes prior to (T0) and after 2, 4 and 12 weeks storage at 4°C (2w, 4w, 12w). The level of significance is indicated by * for 0.05> p >0.01, ** for 0.01> p >0.001 and *** for p <0.001.

**^2^** Multiple loci are ordered according to decreasing sequence similarity
