# Supplementary material for: Novel candidate genes influencing natural variation in potato tuber cold sweetening identified by comparative proteomics and association mapping
Source: BMC Plant Biol. 2013 Aug 7;13:113. doi: 10.1186/1471-2229-13-113 (PMC3750364; doi:10.1186/1471-2229-13-113)
Supplement: Additional file 6: Figure S1 — Positions of SNPs and indels in the amplicons used for association analysis. Position refers to the start codon (A1TG). Primer sequences are underlined. Exons are shaded grey. [file 1471-2229-13-113-S6.docx]

**Supplementary Figure S1**: Positions of SNPs and indels in the amplicons used for association analysis. Position refers to the start codon (A_1_TG). Primer sequences are underlined. Exons are shaded grey.

*KT-InvInh* (PGSC0003DMG200010146)

TCAATAGAATTCCTTACCCGAATTTGATACTTTACCTGCATGTTATTCAGTGTAACACATATTATGTATATATTTTTGTTGATCGTTAAAAAAAAAAAAAAAAGTGTAAAGAGTGACTTAATATTGAAGCTTGATATTAGGTATTTAAAATGAATAAAACTTATTTGAGGTGTCTAAGGCGGTAGTAGGTATTATTTTTTGTTTGCCAAAAATGCCCTTAGCAGCTCTATAAATTGGACATAAACTCATAGCAAGCAAAACACACAAAGAAAGA_1_TGAAGTCGATTAATATTTTGAGTTTCCTTTTGCTTTCAAGTACCCTCTCTTTGGTTGCCTTTGCTCGATCTTTCACTTCTGAGAATCCAATTGTCCTCCCCACAACTTGTCAT**[±GATGAT_114_]**GATAATCTTGTACTCCCTGAAGTTTATGACCAAGATGGCAATCCGCTGAGGATTGGTGAGAGGTACATTATTAAGAATCCTCTCCTCGGG**[C_206_G]**CCGGAGCCGTATACTTGAACAATATTGGAAACCTTCAATGCCCAAACGCCGTGTT**[A_261_G]**CAGCACATGTC**[G_273_A]**ATTCCCCAATTTTTGGGAAAAGGCACGCCCGTC**[A_307_G]**TGTTC**[G_313_A]**TTCGTAAGTCGGAGTCGGAT**[G_334_T]**ATGGTGATGTGGTGCGT**[C_352_G]**TAATGACTG**[C_362_G]**TGTTTATATCAAGTTCTTTGTTAAAACA**[A_391_T]**CAA**[A_395_G][G_396_A]**TTGTGTGTTGACGAAACTGTTTGGAAAGTTAAT**[A/G/C_430_]**ATGAACAGTTGGTGGTAACTGGTGGTAACGTAGGAAATGAAAACGACATCTTCAAGATCAAGAAAACTGA**[C_501_T]**TTGGTGATACGAGGTATGAAAAATGTATACAAGTTACTGCATTGTCCCTCTCATCTT**[C_559_G]**AGTGCAAAAATATCGGCAGCAACTTTAAAAATGGATATCCTCGTCTGGTGACTGTCGATGACGATAAGGACTTTATTCCATTTGTGTTCATCAAGGCGTAGAATGCTAATTAGCTGGCTAGCTTGCAGCTTTTTTAAATAAAGTCGATATATCCTTCTATCGCTCCATGTAATTTAATGTATGCTTATCAATAAATAAACAAGCTAGCAATTATCCTATTACCTTACCTTACCTTCTTCATTCAAAAATACATAAATTTCTTTAGTTTGTGGTTTCTTTTTAAGTTAGGGTTACTTTTGTATTTGTTGTTAATCAGGTCTAGATATCGTTAC

*LapN* (PGSC0003DMG400007831)

GCTTCCTGGTCTTGGCTCAAAAAGGATTGCTCTAGTTGGGCTTGGCTCACCAACATCATCAACTGCTGCTTATCGCTGTTTAGGGGAGGCTGCTGCTGCAGCTGCCAAGTCTGCTCAGGCTAGTAATAT[**C_2704_T]**GC**[C_2707_T]**ATTGCTCTTGCTTCTACGGATGG**[A_2731_G]**CTCTCTGCAGAATC**[G_2746_A]**AAGCTTAGCTCTGCCTCTGCCATAACAACTGGTATC**[C_2783_G]**ATTTTC**[A_2790_G]**TG**[G_2793_A]**TCTTC**[G_2799_A]**CTTAATTCAT**[T_2810_C]**GA**[A_2813_G]**CC**[A_2816_G]**TATTGAGAAACTAA**[±A_2831_]**CTTGGTTGATTTTCGTGAACATTGTAGGAGCTGTGCTGGGGACATTTGAAGATAATAGGTTTAAATCTGAGTCAAAGAAACCAACATTGAAATCTTTGGATATTCTTGGACTGGGGACTGGACCTGAGATAGAGAAGAAAATCAAGTATGCAGCAGATGTCTGTGCAGGTGTTATACTCGGAAGAGAGCTCGTCAATGCACCCGCCAATGTACTTACGCCTGGTTAGTGTTTTTCAATGCATTTCCTTGTTGTCCCTTTTA**[T_3093_A]**TAG**[T_3097_A]**ATG**[A_3101_C]**CTATC**[G_3107_A]**CCACACTAT**[T_3117_C]**AAAATGCCGACTTTTCGCTGCAGCGGTACTTGCTGAAGAGGCCAAAAAGATTGC**[G_3172_A]**TCCACTTATAGCGATGTCTTTTCTGCAAACATCTTGGATGTTGAGCAGTGCAAAGAATTGAAAATGGGATCCTATTTAGCAGTTGCTGCAGCTTCTGCAAATCCTGCTCATTTCATCCATTTGTCTTATAAGCCTAGTAGTGGAGAAATAAAAAAGAAGATAGCCTTGGTTGGAAAGGGATTAACTTTTGACAGGTAATTCTATCTTCTATAAGTTGGAAAAATAGAAATTTGATTTCTGACCTGGCTGC
